# Making the most of the old age: Autumn breeding as an extra reproductive investment in older seabirds

**DOI:** 10.1002/ece3.7431

**Published:** 2021-03-26

**Authors:** Francisco Ramírez, Andre Chiaradia, Danielle A. O'Leary, Richard D. Reina

**Affiliations:** ^1^ Department of Renewable Marine Resources Institut de Ciències del Mar (ICM‐CSIC) Barcelona Spain; ^2^ School of Biological Sciences Monash University Clayton VIC Australia; ^3^ Conservation Department Phillip Island Nature Parks Cowes VIC Australia

**Keywords:** fitness, life‐history strategies, little penguin, marine productivity, reproduction, seabird

## Abstract

The extrinsic and intrinsic factors affecting differing reproductive strategies among populations are central to understanding population and evolutionary ecology. To evaluate whether individual reproductive strategies responded to annual patterns in marine productivity and age‐related processes in a seabird we used a long term (2003–2013), a continuous dataset on nest occupancy and attendance at the colony by little penguins (*Eudyptula minor*) at Phillip Island (Victoria, Australia). We found that concurrent with a secondary annual peak of marine productivity, a secondary peak in colony attendance and nest occupancy was observed in Autumn (out of the regular breeding season in spring/summer) with individuals showing mating‐like behavior. Individuals attending this autumn peak averaged 2.5 years older than those individuals that exclusively bred during spring/summer. Rather than being a naïve response by younger and inexperienced birds misreading environmental cues, our data indicate that the autumn peak attendance is an earlier attempt to breed by older and more experienced penguins. Therefore, we provide strong support for the fundamental prediction of the life‐history theory of increasing investment in reproduction with age to maximize lifetime fitness as future survival prospects diminish and experience increases.

## Graphical abstract

We use an eleven‐year dataset of bird breeding to test the life‐history theory that predicts a relationship between reproductive investment and age. We examined the pattern of attendance at the colony and demographic information of little penguins to detect a peak in breeding activity during autumn, in addition to the usual breeding peak in spring/summer. We found that there was a significant difference in age of birds that bred in one or both these peaks, indicating an increased investment by older birds in accordance with theory predicting their diminished future reproductive potential and capacity to accurately detect environmental cues.

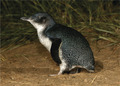

## 1 INTRODUCTION

Life‐history theory describes the fundamental trade‐offs animals must make in allocating limited energetic resources to competing for life‐history functions, particularly reproductive investment and self‐survival (Stearns, 1992). The relative importance of these two competing needs in maximizing an individual's overall fitness will determine whether the animal should invest more resources into the current breeding effort, or store energy for survival and future reproductive potential (Erikstad et al., 1998; Reed et al., 2008). Driven by this trade‐off, the course of evolution has resulted in a myriad of life‐history strategies across the tree of life (Capdevila & Salguero‐Gomez, 2019). Short‐lived species generally display characteristics of fast‐strategists (formerly *r*‐strategies), such as early maturation and a high reproductive effort to maximize the number of offspring produced in each attempt (Nichols et al., 1976), while long‐lived species are typically expected to be slow strategists (formerly *K*‐strategists) and trade‐off their current effort against the probability of adult survival to the following breeding attempt (Bókony et al., 2009; Froy et al., 2013). Strategies for resource allocation to various life‐history traits have been widely investigated at the level of species and with extrinsic (environmental constraints) and intrinsic (e.g., phylogenetical, physiological or demographic constraints) boundary conditions (Stearns, 1992) constraining the available combinations of life‐history traits (Capdevila & Salguero‐Gomez, 2019). In contrast, variations among and within populations have received less attention (Bradley & Safran, 2014; Froy et al., 2013; Martin, 2002; Réale et al., 2010), despite their broad ecological application and potential implications for life‐history and population dynamics (e.g., Hamel et al., 2009; Oppel et al., 2010; Schreiber & Burger, 2001; Wheatley et al., 2008).

Although life‐history strategies are phylogenetically structured (Jombart et al., 2010), individual variations can be found within particular taxa. For example, most seabird species are widely considered as slow strategists (Schreiber & Burger, 2001), but different species can display varying strategies within the life‐history spectrum that ultimately maximize individuals' fitness under varying biological and environmental pressures (Schreiber & Burger, 2001; Skibiel et al., 2013). These strategies can range from the very low reproductive rates (single egg‐clutches, and extended incubation periods) and long generation times (up to decades) of petrels and albatrosses (Froy et al., 2013; Rodríguez et al., 2019), to the short life spans (few years) and the high reproductive investments (many eggs per clutch) of auklets (Jones, 1992; Knudtson & Byrd, 1982). Differences in life‐history strategies have also been observed among populations of the same species as a likely phenotypic response to a given set of environmental conditions or genetically structured differentiation among distant populations (Lahann & Dausmann, 2011; Schultner et al., 2013). Within populations, intrinsic constraints such as an individual's age (a proxy for experience or survival prospects, Froy et al., 2013) may influence the frequency and success of reproductive events over time, by altering the allocation of resources to life‐history functions (Froy et al., 2013; Nisbet & Dann, 2009; Ramírez et al., 2015; Reed et al., 2008; Zimmer et al., 2011).

**FIGURE 1:**
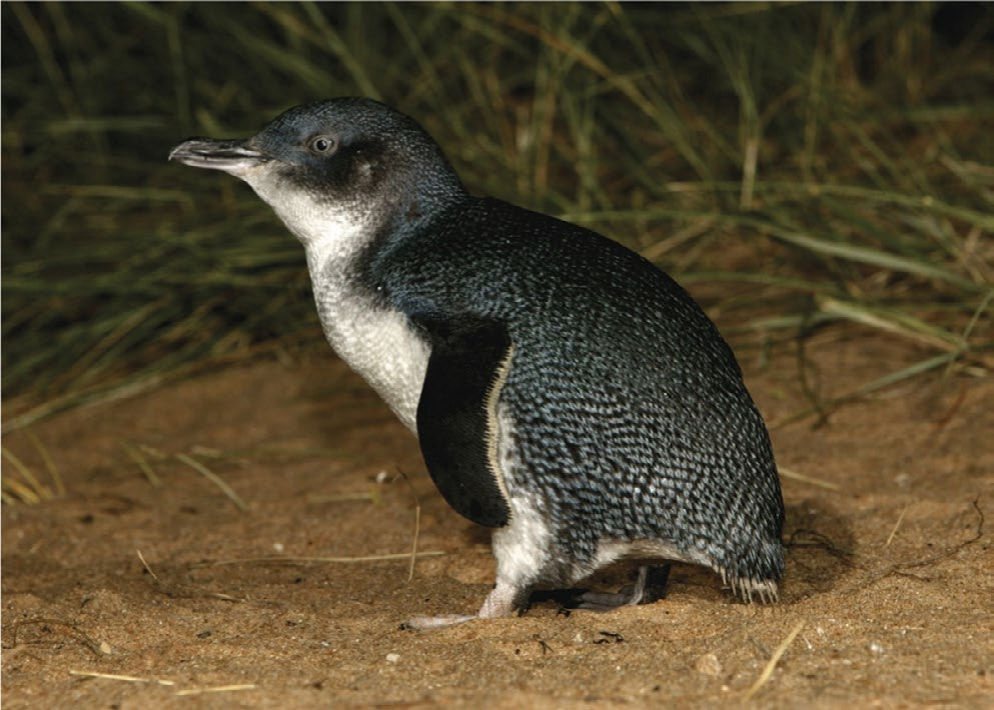
Little penguin (*Eudyptula minor*) from the Phillip Island mega colony. Credit: Phillip Island Nature Parks

The little penguin (*Eudyptula minor*, Figure 1) is an example of a seabird showing fast‐strategists' characteristics compared to other seabird species (Lewis et al., 2012). They have a relatively early age of sexual maturity, usually between two and three years (Priddel et al., 2008), coupled with a relatively short average life span (around 6.5 years, Dann et al., 2005; but 14 years when excluding fledglings, Zimmer et al., 2011, Ramírez et al., 2015). With these particular life‐history traits, there is selective pressure to maximize reproductive output and lifetime fitness (Abraham & Sydeman, 2006), while reducing deleterious impacts on animals' fitness from unfavorable environmental conditions (Ponchon et al., 2014; Saraux et al., 2011). Contrasting reproductive strategies have been documented among little penguin populations. Indeed, the little penguin is one of the few examples of a seabird potentially undertaking multiple breeding events within a year (i.e., additional breeding attempts out of the regular breeding season), although the success of such additional attempts varies among years and between populations as a likely response to environmental forcing factors (Agnew et al., 2014; Gales, 1985; Johannesen et al., 2003). Further, age‐related reproductive strategies of resource allocation to reproduction (i.e., differing use of exogenous resources for egg production by birds of different ages) has also been documented at the individual level (Ramírez et al., 2015), thus pointing to the potential for varying reproductive strategies throughout their life span. As a seabird species that exhibit some characteristics of a fast‐strategist, the little penguin is a very unusual example that cuts across traditional taxonomic life‐history boundaries, making it an interesting case study deserving of a closer inspection.

**FIGURE 2:**
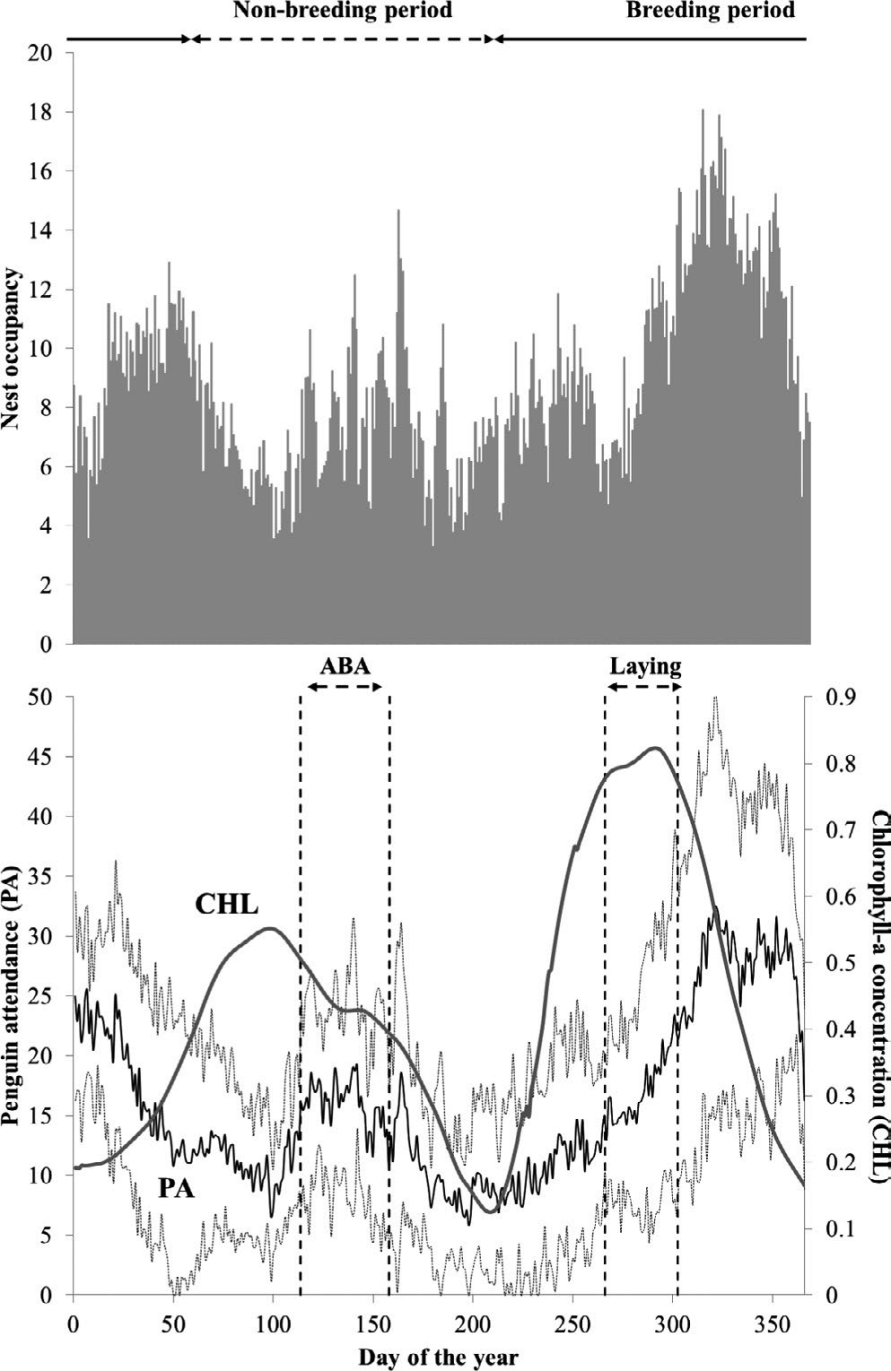
The annual attendance patterns (black line, mean ± *SD*) of little penguins at Phillip Island (Victoria, Australia) over 2003–2013. Vertical dashed lines represent the averaged start and end of the Autumn Breeding Attempt (ABA, see Methods for details on these calculations) and the laying date (Laying, mean ± *SD*) for the regular spring/summer breeding period (2003–2013, Sánchez et al., 2018). Intra‐annual patterns in chlorophyll‐*a* concentration as a proxy for marine productivity (solid gray line) has been taken from (Afán et al., 2015). Gray bars are the average data on the number of nests occupied per day, available for the 2008–2012 period

Little penguins from the mega colony at the Phillip Island (Victoria, Australia) primarily breed during the Austral spring/summer (August to February), when there is an increased in penguin visitation to the colony, leading to the courtship period (Chiaradia & Kerry, 1999). This attendance pattern occurs concurrently with increasing water temperatures in surrounding waters, matching the annual peak of marine productivity in a dynamic marine area that encompass water masses flowing toward, and influencing, little penguin foraging grounds (see details in Afán et al., 2015, see also Figure 2). However, a smaller but prominent peak in attendance has been reported in Autumn. Contrasting with what occurs in other colonies, this attendance was never explored in the context of the breeding cycle in the Phillip Island mega colony (Kowalczyk et al., 2015; Reilly & Cullen, 1981). However, this might still be considered a breeding attempt as patterns of colony attendance to Phillip Island during Autumn resemble the ones for the regular breeding season in spring/summer, and individuals typically display similar mating‐like behaviors (Chiaradia & Kerry, 1999; Salton et al., 2015).

Given that there is a cost in pairing, nest renovation and spending a fasting period ashore (Erikstad et al., 1998), we investigated which extrinsic and intrinsic factors may be associated with this peak of attendance in Autumn and if this should be considered to be a genuine breeding attempt. We combined a long term, continuous dataset of colony attendance and nest occupancy by little penguins at Phillip Island, with environmental information of annual patterns of marine productivity and hence food availability for penguins (Afán et al., 2015). By comparing these patterns among individual little penguins of known age, we examined two alternative explanations for the observed patterns in attendance to the colony in Autumn. The first was those younger and less experienced individuals misread cues that usually trigger breeding in spring/summer (Daniel et al., 2007; Pelletier et al., 2014; Riotte‐Lambert & Weimerskirch, 2013), while the second was those older individuals increase breeding effort to attend in Autumn because of their reduced residual reproductive value and/or their enhanced experience (Froy et al., 2013; Ramírez et al., 2015). We aimed to understand a potential reproductive behavior in this fast‐strategist seabird species and interpret our results in the context of life‐history theory predictions.

## 2 METHODS

### 2.1 Study species and area

The little penguin, *Eudyptula minor*, is the smallest extant penguin species (approx. 1,000 to 1,200 g body mass), typically lays two eggs per clutch (Dann et al., 1995) and is found on the coasts of southern Australia and New Zealand (BirdLife International, 2018). We studied the attendance patterns of mega colony of little penguins at the Phillip Island, Australia (38°30'S, 145°15'E) of approximately 30,000 birds (Sutherland & Dann, 2014).

### 2.2 Fieldwork

Penguins were recorded using an Automatic Penguin Monitoring System (APMS) from 2003 to 2013. The APMS identifies individual penguins with TIRIS^TM^ and Allflex^TM^ passive integrated transponders and records the date, time and body mass (g) of penguins as they move in and out of the colony across a weighing platform placed on a single main track within the nesting area (Chiaradia & Kerry, 1999). In addition to the APMS data, as part of a long‐term research program, nest occupancy was monitored using a handheld transponder‐reader during the 2003 to 2013 spring/summer breeding seasons (September‐February) and 2008 to 2012 nonbreeding seasons (March‐July). During the spring/summer breeding season, the colony was checked three times a week for the presence of breeding penguins, eggs or chicks and phases of reproduction. Outside this period, the site was visited once per week. Prefledgling chicks and any unmarked adult breeding penguins were injected with Allflex^TM^ transponder tags (Chiaradia & Kerry, 1999) and bill depth measured to determine the sex of adults (Reilly & Cullen, 1981).

### 2.3 Data processing and analyses

The dataset downloaded from the APMS was filtered before analysis. Only penguins marked with TIRIS^TM^ or Allflex^TM^ transponder numbers and crossed the APMS were included in the study. Crossings of the same individual that were less than 40 min apart were removed to reduce the effect of pseudo‐replication. Individual body mass under 700 g or over 1,700 g were removed from the analysis (modified from Salton et al., 2015). As many penguins use the APMS to enter the colony, but do not always exit via the same route, only incoming APMS crossings were used in the analyses.

Given that birds are present in the colony at all times of the year, we followed Chiaradia and Kerry (1999) and considered that the peak in autumn attendance started when the number of penguins that entered the colony in a day was at least twice the minimum number recorded in March (the previous, postmolt period, Figure 2) for at least 5 successive days. The peak ended when the number of penguins entering the colony per day declined by at least 50% from the maximum number recorded during the immediately preceding period, and no further peak in APMS incoming crossings was detected for at least 14 days.

We calculated the mean ages and proportion of male and female penguins that both bred during the regular spring/summer breeding season and also attended the colony during the autumn peak period in any given year from 2003 to 2013. We compared the age of these penguins with those breeding only during spring/summer. The age of most birds was calculated in the year an individual was marked with a transponderat fledging. For the few individual penguins that were marked as an adult, we added three years to their calculated age to allow for the time taken for a fledgling to become sexually mature and return to the colony as a breeding adult (Nisbet & Dann, 2009).

A paired *t*‐test was used to detect differences in the mean age between penguins that attended the colony during both Autumn and the regular spring/summer breeding seasons and those that only bred in spring/summer across years. Analyses were conducted in R (R Core Team, 2019).

## 3 RESULTS

The study included 332 marked individual penguins of known age, with approximately 270,500 colony attendances (i.e., incoming APMS crossings) throughout the 10 breeding seasons of this study.

The number of penguins coming ashore fluctuated throughout the year with colony attendance pattern during the autumn peak that had a similar pattern to the regular spring/summer breeding season (Figure 2). We observed two distinct peaks in the number of penguins entering the colony each year in Autumn (Figure S1). The first peak was the longest, starting on average on 27th April (ranging from 16th April to 19th May) and lasting for about 17 days (mean ± *SD*: 16.6 ± 3.8 days, *n* = 11). A clear decline followed this initial peak in the number of penguins entering the colony, which lasted for 10.3 ± 4 days. The second peak started on average on 24th May (8th May to 28th June) and lasted 12.5 ± 2.7 days. The annual pattern of two peaks and a trough in attendance during Autumn was consistent with the time series, except for 2012 when this pattern appeared to repeat, resulting in three or four distinct peaks in the number of penguins entering the colony (Figure S1).

Nest occupancy generally followed a similar, slightly delayed pattern to that of colony attendance, increasing during the regular spring/summer breeding season as well as peaking during Autumn of each year (Figure 2). These patterns were also similar to the intra‐annual variability in marine productivity, as indicated by chlorophyll‐*a* concentration (Figure 2). Seasonal patterns in marine productivity at this area have been described in Afán et al. (2015), but are included here for illustrative purposes. The main peak in marine productivity occurs in early spring with the increase in light penetration within a nutrient‐rich water column after winter mixing. This main peak matches with the laying date for the regular spring/summer breeding attempt (see Afán et al., 2015 for details and Figure 2). A secondary peak in marine productivity occurs during the Autumn a few weeks before the autumn peak attendance (Figure 2).

On average, 76% (SE = 6.2) of marked adult birds breeding during the regular spring/summer season also attended the colony during the autumn peak attendance (Table 1). There was no significant difference between the number of males and females who attended the colony during the autumn peak attendance (*t* = 0.85, *p* = .42) (Table 1). Penguins that both bred in spring/summer and attended the colony during the autumn peak attendance were on average 2.5 years older than those penguins that only bred in the spring/summer in a given year (t = 5.2, *p* < .001) (Table 1).

**TABLE 1:** The number, proportion, sex and average age of little penguins that bred during the regular spring/summer breeding period, compared to those that both bred during spring/summer and also attended the colony during the autumn breeding attempt (ABA)

	Penguins at the spring/summer breeding season	Proportion of penguins at spring/summer breeding season & ABA			Average age of penguins	
		Total	Females	Males	spring/summer breeding season	spring/summer breeding season & ABA
2003	94	0.74	0.7	0.79	6.4	7.5
2004	117	0.65	0.58	0.72	3.16	7.6
2005	110	0.77	0.67	0.64	4.1	7.4
2006	113	0.79	0.81	0.77	3.8	7.3
2007	98	0.77	0.76	0.77	6	7.6
2008	144	0.79	0.79	0.79	4.7	7.9
2009	125	0.66	0.68	0.65	7	8.4
2010	130	0.82	0.78	0.85	5.08	9.2
2011	126	0.8	0.81	0.8	5.3	9
2012	132	0.86	0.85	0.87	7.8	9.2
2013	117	0.75	0.78	0.73	10.1	9.5

## 4 DISCUSSION

The peak of penguin attendance in Autumn followed a similar pattern to that of the penguins breeding in spring/summer (Chiaradia & Kerry, 1999). Additionally, the environmental factors triggering reproduction during the regular spring/summer breeding season were also present in Autumn and may trigger the autumn peak attendance (Afán et al., 2015). These data, the presence of an approximately equal number of males and females and the characteristic breeding‐like behavior of the birds leads us to conclude that the autumn peak attendance is an earlier attempt to breed, that we term the “autumn breeding attempt” (hereafter abbreviated to ABA). During the ABA, individuals attending the colony also reproduced during the spring/summer period and were 2.5 years older than penguins exclusively breeding in the spring/summer breeding period. Rather than being a naïve response by younger and inexperienced individuals misreading environmental cues, we argue that the older, more experienced penguins use the ABA to maximize their lifetime fitness through a higher reproductive investment as their future survival prospects diminish.

Throughout their lifetime, individuals are exposed to varying pressures or constraints (i.e., "boundary conditions"; Stearns, 1992), and they can attempt to maximize their lifetime fitness by adapting their resource allocation strategies (Nichols et al., 1976). As individuals age and their residual reproductive value decreases and their experience increases, resource allocation to reproduction is expected to rise, sometimes at the expenses of their own survival and, hence, of their future reproductive attempts (Froy et al., 2013; Nisbet & Dann, 2009; Ramírez et al., 2015). Therefore, older seabirds are more likely to try to increase their breeding output by starting reproduction earlier in the year or by engaging in additional breeding attempts within a year (Limmer & Becker, 2010). Based on a long term (2003–2013) and large dataset on reproductive attempts for little penguins of known age (270,500 colony attendances for 332 marked individuals), we provide strong support for this fundamental prediction life‐history theory. In particular, we show that birds that attempted to breed in Autumn were on average 2.5 years older than those that exclusively reproduced during spring/summer breeding period. Owing to their relatively short life span (Dann et al., 2005; Ramírez et al., 2015; Zimmer et al., 2011) this represents a substantial difference in individuals' ages between these two groups, consistent with a strategy for increasing overall reproductive output and lifetime fitness (Abraham & Sydeman, 2006).

Additional breeding attempts outside the regular breeding period are rare among seabirds and typically occurs in species that have short life spans and live in highly variable environments, where flexible reproductive strategies are required to benefit from conditions that vary over a relatively brief timescale (Abraham & Sydeman, 2006; Schroeder et al., 2009). This type of behavioral and reproductive plasticity may also reduce the impact of environmental variables on seabirds' fitness under unfavorable conditions (Ponchon et al., 2014; Saraux et al., 2011). In turn, fluctuations in environmental variables during favorable conditions, coupled with a range of internal cues or constraints such as age (Daniel et al., 2007; Nisbet & Dann, 2009; Pelletier et al., 2014), will determine the probability of a given individual engaging in multiple breeding attempts (Johannesen et al., 2003).

Afán et al. (2015) demonstrated that marine productivity patterns (using chlorophyll‐*a* concentrations as a proxy) in the waters surrounding the Phillip Island penguin mega colony were more predictable than previously thought (see details in Afán et al., 2015). In particular, these authors showed that two different annual peaks in marine productivity consistently occur in spring (the main peak) and Autumn (a secondary peak), matching little penguins' regular breeding and ABA periods respectively. This could potentially allow individuals to synchronize the high energy‐demanding period of reproduction with seasonal pulses in food availability (Afán et al., 2015; Durant et al., 2005; Reed et al., 2006). In turn, this would also imply the ability of individuals to use environmental cues to anticipate favorable conditions, necessary because of the time required to accomplish breeding preparations (e.g., gonadal preparation Nager, 2006; mate selection Naves et al., 2006; or storage of endogenous resources Ramírez et al., 2015). Sea temperature has been suggested as a likely driver of seabirds' reproductive phenology (Weimerskirch et al., 2001), and little penguins in particular (Afán et al., 2015), when rising sea temperature was a clear precursor signal of the temporally lagged (seven‐week delay) main annual productivity peak. Similarly, the secondary peak in annual productivity occurred a few weeks after the fall in local sea temperatures, which typically peak in late March (Afán et al., 2015). Thus, seasonal variations (both upwards and downwards) in local sea surface temperature could be used by penguins as the environmental cue for both the regular spring/summer breeding attempt and the ABA. The decision of the bird to engage with an additional breeding attempt in response to the environmental cue would be additionally influenced by its age and foraging opportunities.

Overall, trends of colony attendance and nest occupancy observed during ABA resembled well‐established patterns that have been reported during the spring/summer breeding season later in the year (Chiaradia & Kerry, 1999; Reilly & Cullen, 1981). Indeed, the peaks and troughs in colony attendance during ABA corresponded with the three main phases of the spring/summer breeding season; “courtship,” “prelaying exodus” and the “laying” phases (Chiaradia & Kerry, 1999). The similar number of males and females that attended the colony during Autumn was also consistent with them forming pairs for breeding. Coupled with the relatively favorable environmental conditions from the secondary peak in marine productivity, this would point to ABA as an actual breeding attempt. Multiple breeding seasons within a single year are known in African penguins (Crawford et al., 1999) and appear to be associated with a variable pattern of gonadal regression (Mafunda et al., 2021). The gonadal hormones and development/regression patterns of little penguins are unknown, but our results suggest that a similar situation might occur in this species. Further investigation on this phenomenon may provide key insights on the physiology of reproductive cycles of penguins and their capacity to breed at different times of year.

The ABA rarely eventuates in the production of offspring in the Phillip Island mega colony (Reilly & Cullen, 1981), and thus differs from similar behaviors observed in other little penguin colonies (Kowalczyk et al., 2015) and even other penguin species, which regularly produce successful clutches in Autumn or multiple clutches in a year (Johannesen et al., 2003; Paredes et al., 2002). We suggest that reproductive failure in Autumn at the Phillip Island colony might result from suboptimal environmental conditions. The smaller peak in marine productivity and food availability, occurring in Autumn may be not enough to sustain a full breeding event during ABA at this mega colony at Phillip Island. Additionally, the intense intraspecific competition due to the large size of the Phillip Island colony (Sánchez et al., 2018) may act to reduce further the amount of high‐quality prey resources available within the local foraging range, therefore negatively affecting the breeding performance of individuals (Forero et al., 2002; Ramírez et al., 2014). In contrast, in smaller colonies, where food availability per capita is higher, little penguins can engage in additional and successful reproductive events throughout the year despite intraspecific competition (Gales, 1985; Johannesen et al., 2003; Kowalczyk et al., 2014, 2015).

While larger population size may lower the probability of successful additional breeding attempts; this may not deter individuals from maximizing their overall reproductive output by attempting multiple clutches under favorable conditions in response to environmental cues (Perriman et al., 2000). Little penguins are less slow strategists than most seabirds (Chiaradia et al., 2016); for example, they have high adult mortality of 40% (Dann & Cullen, 1990), and can have two and sometimes three clutches in one given regular breeding season (Agnew et al., 2014; Johannesen et al., 2003). These are indicative of opportunistic breeding behavior. Double clutches are overlapping events, that is, a second reproductive effort (clutch of eggs) begins while the first reproductive effort (unfledged chicks) are still in being cared for in the breeding colony (Agnew et al., 2014; Gales, 1985; Johannesen et al., 2003). The ABA event in our study here differs from this pattern because it is a single isolated breeding attempt that ends in Winter, with an intervening break before breeding then resumes in Spring/Summer. Thus, any breeding attempt would be an opportunity to maximize individuals' overall breeding potential and lifetime fitness by trying to lay additional successful clutches within a year (Agnew et al., 2014; Johannesen et al., 2003). This must be particularly true for aging individuals, that may display less "reproductive restraint" due to their low residual reproductive potential in later years (Bradley & Safran, 2014; Froy et al., 2013; Ramírez et al., 2015). Indeed, breeding penguins that did attend the colony during Autumn were on average 2.5 years older than those that only bred in the spring/summer of a given year. This underlying mechanisms of age‐related patterns agree with the ability of aging little penguins to produce a second clutch within the same, regular breeding attempt during the spring/summer period (Agnew et al., 2014). In this two‐egg clutch seabird, it has been suggested that second laid‐eggs would be of lower quality but viable, potentially resulting in successful fledging if food resources are plentiful at chick‐rearing (Ramírez et al., 2015). Analogously, ABA could be an additional reproductive attempt to maximize reproductive output in years where environmental conditions may be favorable. Currently, little penguins breeding in Phillip Island are advancing their breeding phenology as a likely response to climate‐driven environmental changes (Keogan et al., 2018). Within this scenario, ABA may become more frequent within the studied population.

Alternatively, older penguins may attend the colony during ABA because they are more experienced, so individuals can better detect and respond to subtle environmental cues that signal the potential of an additional reproductive attempt (Nevoux et al., 2007, 2010; Pyle et al., 1991). Furthermore, age‐related experience has also been linked to an earlier seasonal onset to reproduction, increased clutch sizes and improved foraging skills, resulting in more frequent and successful breeding attempts compared to younger birds (Limmer & Becker, 2010; Nisbet & Dann, 2009; Zimmer et al., 2011). Breeding performance of little penguins in the Phillip Island mega colony typically increases from first breeding up to 7 years of age, then plateaus and subsequently declines after 16 years (Nisbet & Dann, 2009). This pattern agrees well with age‐related trends in foraging skills and individuals' body condition, whereby more experienced and better‐conditioned middle‐aged (5–10 years) little penguins forage better than inexperienced younger (3–4 years) and poorer‐conditioned but experienced older (11–14 years) individuals (Zimmer et al., 2011). The average age of individuals that attended both the ABA and the regular, spring/summer breeding season in our study largely fell within the mid‐to late‐middle‐aged category, suggesting the potential role of an individuals' experience in shaping resource allocation strategies to reproduction. However, Ramírez et al. (2015) provide evidence suggesting that older little penguin females might be preparing themselves well in advance for breeding by accumulating larger reserves during the prelaying period and relying more on endogenous reserves for clutch production. Thus, age‐related individual reproductive strategies may be associated with lower survival prospects, and the low residual reproductive potential in later years, rather than advantages in foraging associated with experience (Bradley & Safran, 2014; Ramírez et al., 2015), or at least some combination of the two.

Previous studies on little penguins have reported an attendance peak at the Phillip Island mega colony in Autumn (Salton et al., 2015). Here we explored that autumn pattern further in an interdisciplinary approach that combines biological data (colony and nest attendance) with environmental information (trends in sea temperature and patterns in marine productivity) within an ecological/evolutionary framework that relies on the consistent evidence for the existence of age‐related increments in individuals' reproductive effort (Beamonte‐Barrientos et al., 2013; Christians, 2002; Ramírez et al., 2015). Although we could not identify the ultimate cause underlying resource allocation strategies to reproduction in little penguins, we provide strong evidence pointing to age and age‐related "boundary conditions" (i.e., experience and senescence) as important drivers of individual strategies based on single versus. double brooding. Although additional breeding attempts appear to be largely unsuccessful in our study colony, we speculate that such strategies would be directed to optimize individual investment in fecundity versus. survival throughout their life span and according to the varying intrinsic conditions of increasing experience or decreasing residual reproductive value that constrain the available combinations of these life‐history traits (Capdevila & Salguero‐Gomez, 2019). Accordingly, this study is a useful contribution to understanding of life‐history trade‐offs in resource allocation to reproduction in birds.

## CONFLICT OF INTEREST

None declared.

## AUTHOR CONTRIBUTIONS

AC, DO and RR conceived the ideas and designed the methods for the project. AC and DO collected the data, AC, DO, FR and RR analyzed the data. AC, FR and RR led the writing of the manuscript. All authors gave approval for publication and declare that they had no conflict of interest.

## Supporting information

Fig S1

## Data Availability

Raw and analyzed data supporting the results are stored at the official repository of Monash University and are available at https://doi.org/10.26180/12847661.
