# Supplementary material for: Making the most of the old age: Autumn breeding as an extra reproductive investment in older seabirds
Source: Ecol Evol. 2021 Mar 26;11(10):5393–401. doi: 10.1002/ece3.7431 (PMC8131812; doi:10.1002/ece3.7431)

SUPPORTING INFORMATION

**Figure S1.** The annual attendance patterns of little penguins at Phillip Island (Australia) over the 2003-2013 period. Grey bars are data on the number of nests occupied per day, available for 2008-2012.


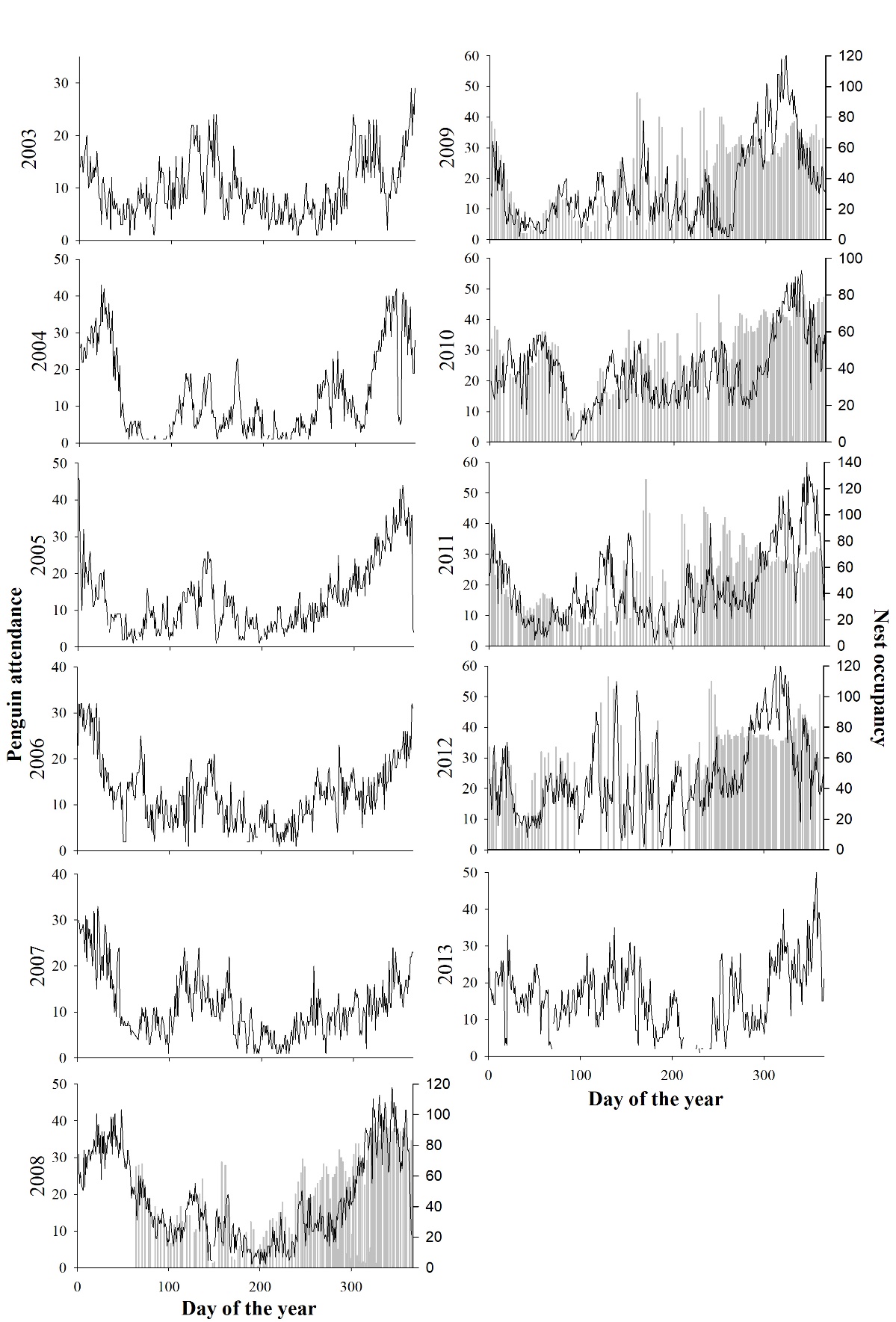

Supplement: Supplementary file 1 — Fig S1 [file ECE3-11-5393-s001.docx]
